# Vitamin D deficiency causes rickets in an urban informal settlement in Kenya and is associated with malnutrition

**DOI:** 10.1111/mcn.12452

**Published:** 2017-05-03

**Authors:** Kelsey D. J. Jones, C. Ulrich Hachmeister, Maureen Khasira, Lorna Cox, Inez Schoenmakers, Caroline Munyi, H. Samira Nassir, Barbara Hünten‐Kirsch, Ann Prentice, James A. Berkley

**Affiliations:** ^1^ KEMRI‐Wellcome Trust Research Programme Kenya; ^2^ Section of Paediatrics and Centre for Global Health Research Imperial College London UK; ^3^ Baraka Health Centre German Doctors Nairobi Nairobi Kenya; ^4^ MRC Human Nutrition Research Elsie Widdowson Laboratory Cambridge UK; ^5^ Department of Medicine, Faculty of Medicine and Health Science University of East Anglia Norwich UK; ^6^ MRC Keneba The Gambia; ^7^ Nuffield Department of Clinical Medicine University of Oxford Oxford UK

**Keywords:** acute malnutrition, Africa, poverty, rickets, urbanization, vitamin D

## Abstract

The commonest cause of rickets worldwide is vitamin D deficiency, but studies from sub‐Saharan Africa describe an endemic vitamin D‐independent form that responds to dietary calcium enrichment. The extent to which calcium‐deficiency rickets is the dominant form across sub‐Saharan Africa and in other low‐latitude areas is unknown. We aimed to characterise the clinical and biochemical features of young children with rickets in a densely populated urban informal settlement in Kenya. Because malnutrition may mask the clinical features of rickets, we also looked for biochemical indices of risk in children with varying degrees of acute malnutrition. Twenty one children with rickets, aged 3 to 24 months, were identified on the basis of clinical and radiologic features, along with 22 community controls, and 41 children with either severe or moderate acute malnutrition. Most children with rickets had wrist widening (100%) and rachitic rosary (90%), as opposed to lower limb features (19%). Developmental delay (52%), acute malnutrition (71%), and stunting (62%) were common. Compared to controls, there were no differences in calcium intake, but most (71%) had serum 25‐hydroxyvitamin D levels below 30 nmol/L. These results suggest that rickets in young children in urban Kenya is usually driven by vitamin D deficiency, and vitamin D supplementation is likely to be required for full recovery. Wasting was associated with lower calcium (*p* = .001), phosphate (*p* < .001), 25‐hydroxyvitamin D (*p* = .049), and 1,25‐dihydroxyvitamin D (*p* = 0.022) levels, the clinical significance of which remain unclear.

## 1 INTRODUCTION

Rickets is a disease of childhood that occurs due to failure of regulated chondrocyte apoptosis and deficient mineralisation at the growth plates of long bones (Pettifor, 2003; Sabbagh et al*.*, 2005). Children develop a wide variety of skeletal abnormalities that vary according to age and developmental stage, including wrist widening, bowed legs, craniotabes with frontal bossing and delayed fontanelle closure, costochondral swelling, and spinal deformity (Elder & Bishop, 2014). Impaired linear growth and developmental delay are common. Younger infants may present with symptomatic hypocalcaemia (Ladhani et al., 2004; Hogler, 2015).

Worldwide, the commonest cause of rickets is vitamin D deficiency, which leads to secondary hyperparathyroidism and progressive renal phosphate loss (Glorieux & Pettifor, 2014). Vitamin D can be synthesised in the skin in a process that is dependent on sunlight exposure, or derived from a relatively limited range of dietary sources, including egg yolk and oily fish. Deficiency is usually the result of inadequate sun exposure, which may occur due to living at mid‐ or high‐latitude. In tropical or subtropical climes, there is generally abundant exposure to sunlight, but vitamin D deficiency may arise in association with risk factors such as darker skin pigmentation, atmospheric pollution, and covering skin for religious or cultural reasons (Baroncelli et al*.*, 2008; Elder & Bishop, 2014; Trilok Kumar et al., 2015). However, in such settings, vitamin D deficiency may not be the only cause of rickets. Studies from several sub‐Saharan African countries have implicated chronic dietary calcium deficiency in the pathogenesis of rickets and demonstrated nutritional cure with calcium supplementation alone (Thacher et al., 1999; Pettifor, 2004; Prentice et al., 2008; Braithwaite et al., 2016). The aetiology may be multifactorial, because calcium deficiency tends to exacerbate the impact of marginal vitamin D status, and may further interact with other nutritional deficiencies, such as of iron (Braithwaite et al., 2012; Pettifor, 2014).

A recent report highlighted the existence of a significant burden of rickets amongst children living in an informal settlement in Nairobi, Kenya (Edwards et al., 2014). Most children with clinically‐defined rickets were younger than 2 years, and rickets was associated with acute malnutrition and developmental delay (Edwards et al., 2014). In this paper, we describe a similar group of children presenting to a primary care facility in Mathare, another of Nairobi's informal settlements. Via detailed dietary and biochemical evaluation, we provide evidence that deficiencies in both calcium and vitamin D are likely to play a role in the aetiology of rickets in urban Kenyan settings. Because malnutrition may mask the clinical features of rickets, we also looked for biochemical features indicating rickets risk in children with varying degrees of acute malnutrition. To this end, we demonstrate an association between subclinical vitamin D deficiency and severe wasting.

**Key messages**

- In urban Kenya, rickets in young children is usually related to vitamin D deficiency.
- Rickets occurs in infancy, often alongside malnutrition.
- Children with acute malnutrition commonly have low vitamin D status without clinically evident rickets.

## 2 METHODS

### 2.1 Study design

The study was designed primarily as a case–control study comparing children with rickets (cases) to children without rickets or acute malnutrition (controls). Additionally, children with severe and moderate acute malnutrition (SAM and MAM) were compared to controls.

### 2.2 Setting

The study took place between July 2013 and April 2014 at the Baraka Health Centre (BHC), Mathare, Nairobi. BHC is supported and administered by the nongovernmental organisation “German Doctors Nairobi” and provides free healthcare to children aged less than 5 years (http://www.germandoctorsnairobi.co.ke/). Mathare is the second largest urban informal settlement in Kenya. It is home to at least 200,000 people, who mainly live in overcrowded iron‐sheet housing with limited access to safe water and sanitation facilities (Muungano Support Trust, Slum Dwellers International, University of Nairobi, University of California, Berkley, 2012). Most residents engage in casual work or run small businesses and tend to work long hours. Provision for childcare is limited and represents a significant household expenditure for many families (Muungano Support Trust, Slum Dwellers International, University of Nairobi, University of California, Berkley, 2012). Breastfeeding is almost universal and persists beyond 12 months in the majority of cases, although provision of weaning and complementary foods occurs much earlier than the World Health Organization‐recommended 6 months (Kimani‐Murage et al., 2011).

Nairobi is at latitude 1.3°S. It has a subtropical highland climate with two rainy seasons (March–May and October–December). The mean number of hours of sunshine per day peaks in February at 9.5 h/day, dropping to 4.1 hr/day in August (average 6.8 hr/day; World Meterological Organization, 2010). For comparison, The Gambia, where calcium dependent rickets is prevalent, averages 8.0 hr/day (Prentice et al*.*, 2008).

### 2.3 Participants

Children with rickets were recruited from among those presenting to BHC. Inclusion criteria for cases were age 3 to 24 months, with clinically diagnosed rickets, based on the presence of one or more of wrist widening, rachitic rosary, swollen knees, bow legs, or bone pain on walking (Thacher et al., 2002). Where wrist widening was the only feature, clinical evaluation was supplemented with radiographic assessment (right wrist X‐ray), and children were only included in the presence of clear radiologic features consistent with rickets. Controls without clinical features of rickets or acute malnutrition and a further group of children without clinical features of rickets with SAM and MAM were recruited from among those self‐presenting to BHC or via a program of active case finding in the community by local community health volunteers. SAM was defined on the basis of mid‐upper arm circumference (MUAC) less than 115 mm or the presence of bilateral lower limb oedema (kwashiorkor), MAM was defined on the basis of MUAC 115 mm to 124 mm, and nonmalnourished controls had MUAC greater than 124 mm.

Children were excluded (from all of the groups) if they required emergency medical care, if they were being treated for tuberculosis, if they had a fracture within the preceding 3 months, and in the absence of informed parental consent for participation. Children were also excluded if they were known or found to have HIV infection, or if they were younger than 12 months old and known to be HIV‐exposed.

### 2.4 Study procedures

Participants were clinically assessed by a senior clinician (KDJJ or CUH). Demographic characteristics were recorded including anthropometric measurements. A contextually‐relevant, recall‐based food frequency questionnaire for the main carer was developed based on the most frequently consumed sources of calcium and phosphate in this setting, in order to estimate intake of calcium and phosphate beyond that derived from breastfeeding over a 1 week period. The questionnaire was administered by a trained field worker in Kiswahili or English as preferred. Nutrient intakes were calculated with reference to the United States Department of Agriculture's *National Nutrient Database for Standard Reference (Release 28* *)* (United States Department of Agriculture, 2015). Right wrist X‐ray changes were graded according to the method of Thacher et al*.*, whereby Grade 1 rachitic changes were widened growth plate and irregularity of metaphyseal margins, and Grade 2 were metaphyseal concavity and fraying of margins (Thacher et al*.,* 2000).

Venous blood was taken on the same day. Serum calcium, inorganic phosphate, albumin, and alkaline phosphatase (ALP) were measured by a commercial Good Clinical and Laboratory Practice‐accredited laboratory in Nairobi (Pathologists Lancet, Kenya). Aliquots of plasma and serum were stored at −80°C prior to shipment to the laboratories at MRC Human Nutrition Research, Cambridge, UK. There, serum was analysed for 25‐hydroxyvitamin D (25[OH]D; Liaison™ chemiluminescent immunoassay, DiaSorin S.p.A.), C‐reactive protein (CRP); RCRP immunoturbidimetric method on Dimension Xpand™, Siemens Healthcare Diagnostics), alpha‐1 acid glycoprotein (AGP; Immunoturbidimetric method on Dimension Xpand™, Siemens Healthcare Diagnostics) and intact parathyroid hormone (PTH; Immulite™ solid phase chemiluminescent ELISA, Siemens Healthcare Diagnostics). EDTA plasma was analysed for 1,25‐dihydroxyvitamin D (1,25[OH]_2_D; RIA, IDS Ltd,). All assays were conducted in accordance with standard operating procedures and ISO 9001:2008, including quality control vsamples alongside study samples and subscribing to national external quality assessment schemes. The Liaison chemiluminescent immunoassay for total 25(OH)D measures 25(OH)D2 and 25(OH)D3. The assay has been standardised against the international reference method, isotope‐dilution liquid chromatography–tandem mass spectrometry (ID‐LC–MS/MS), and shown to give results on average very close to the international reference methods (approximately 3% higher; Cashman et al., 2016).

### 2.5 Statistical analysis

Analysis was performed in STATA Version 12.0. Weight‐for‐length (WLZ), length‐for‐age (LAZ), weight‐for‐age and occipitofrontal circumference‐for‐age z‐scores were calculated using WHO Anthro Version 3.2.2 STATA macros. Although rickets can directly impact on height due to lower limb skeletal abnormalities, these were rare in our population so the use of height‐based anthropometric variables was assumed to be valid. Serum calcium concentration was adjusted for serum albumin throughout, using the formula Calcium_adjusted_ = Calcium_measured_ + 0.8 × (40–albumin [g/L]), where the adjusted value may be considered a proxy for biologically active calcium (Association for Clinical Biochemistry & Laboratory Medicine, 2015). We used a 25(OH)D lower limit for bone health of 30 nmol/L, above which 25(OH)D‐deficiency rickets is considered unlikely (Munns et al., 2016). Biochemical, demographic, and anthropometric variables for children in each of the rickets, SAM, and MAM groups were compared to controls: Mann–Whitney U or Fisher's exact tests were used to test differences between groups. Outputs of linear regressions are presented adjusted for a priori potential confounders: age, CRP, or AGP, where these were associated with the dependent variable in univariate analyses. No formal sample size calculation was performed: patients presenting with rickets were recruited opportunistically over a 9‐month period, and we planned to recruit a similar number of controls and participants with SAM and with MAM. Statistical correction for multiple comparisons was not performed.

### 2.6 Ethics

All participants enrolled in the study had individual written informed consent provided by a parent or guardian. The study was approved by the Kenya Medical Research Institute's Ethical Review Committee prior to initiation (2481). All children who presented to BHC or were identified through community screening with rickets, SAM, MAM, or intercurrent illnesses were provided with free medical care whether or not they were enrolled in the study.

## 3 RESULTS

Eighty‐four children were recruited to the study: 21 cases of rickets, 22 controls who were nonrachitic and nonacutely malnourished, 21 children with SAM (of whom nine had kwashiorkor), and 20 with MAM. Monthly recruitment of children with rickets was similar throughout the study period.

### 3.1 Clinical and biochemical phenotype of rickets

Most of the children with rickets had wrist widening and rachitic rosary as opposed to lower limb features (Table 1). Developmental delay, acute malnutrition, and stunting were common (Table 1).

**Table 1:** Clinical features of rickets

Clinical features		*n* (%)
Major features	Wrist widening	21 (100)
	Rachitic rosary	19 (90)
	Swollen knees	4 (19)
	Bowed legs	3 (14)
	Bone pain on walking	1 (5)
Minor features	Open Fontanelle	19 (90)
	Double malleoli	11 (52)
	Harrison's groove	10 (48)
	Lower arm bending	3 (14)
	Developmental delay	11 (52)
Comorbidity	Fever	5 (24)
	URTI	13 (62)
	Pneumonia	2 (10)
	Acute watery diarrhoea	3 (14)
Nutritional status	SAM	4 (19)
	MAM	11 (52)
	Stunting	13 (62)

*Note*. Major features are defined in line with Thacher et al. (2002). Developmental delay was based on parental report. Pneumonia and acute watery diarrhoea were diagnosed in line with WHO guidance (World Health Organization, 2005). SAM and MAM were diagnosed on the basis of MUAC and/or presence of oedema, stunting was diagnosed if height/length‐for‐age z‐score was ≤2. MAM = moderate acute malnutrition; SAM = severe acute malnutrition; URTI = upper respiratory tract infection.

**Figure 1:**
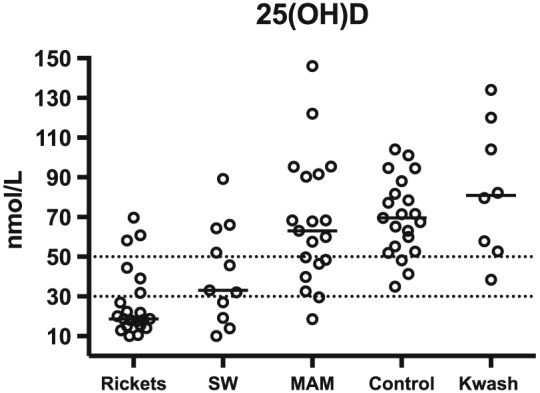
Serum 25‐hydroxyvitamin D (25(OH)D) levels in children with clinically defined rickets, or without rickets but with severe wasting (mid‐upper arm circumference [MUAC]<115 mm), moderate wasting (MUAC 115 to 124 mm), kwashiorkor (Kwash), or community controls. Dotted lines are at 30 nmol/L (taken to indicate likely deficiency) and 50 nmol/L (30 to 50 nmol/L is considered insufficient) (Munns et al*.*, 2016)

**Figure 2:**
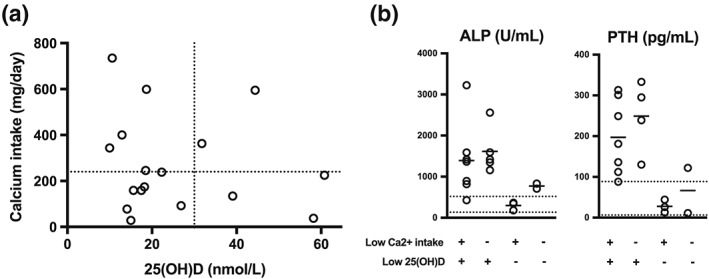
(a) calcium intake and serum 25(OH)D levels from children with clinically defined rickets (from 17 of 21 children where both pieces of data were available). Dotted lines are at 25(OH)D = 30 nmol/L (taken to indicate likely deficiency), and calcium intake 240 mg/day (taken as a representative lower reference nutrient intake [United Kingdom Department of Health, 1991; Munns et al., 2016]). (b) alkaline phosphatase and parathyroid hormone according to adequacy of calcium intake and serum 25(OH)D. Dotted lines are reference range, bars indicate the mean

Children with rickets were relatively hypocalcaemic, hypophosphataemic, and had low 25(OH)D, compared to controls (Table 2). ALP and PTH were elevated (Table 2). Rickets was not associated with elevated 1,25(OH)_2_D (as has been reported in calcium deficiency rickets), and there were no detectable differences in inflammatory markers (CRP and AGP) or in dietary intake of either calcium or phosphate between children with rickets and controls (Table 2).(Pettifor & Prentice, 2011). Although some children with rickets had very low 25(OH)D, six (29%) of them had levels above 30 nmol/L (Figure 1). Twenty‐five(OH)D was not associated with age (supplementary Figure 1). To explore the cause of rickets in these children, we plotted nonbreastfeeding dietary calcium intake against serum 25(OH)D. Most of the children with rickets in the context of higher serum 25(OH)D had calcium intakes below a representative lower reference nutrient intake of 240 mg/day, but this proportion (three out of five, 60%) was not significantly different from the proportion of control children who had low calcium intakes (82%, *p* = .58; Figure 2A) (United Kingdom Department of Health, 1991). Those without low 25(OH)D had substantially less induction of ALP and PTH (Figure 2B).

**Table 2:** Nutritional and biochemical features

	**Rickets (R)**	**Control (C)**	***p* (R vs C)**	**SAM**	**MAM**
*n*	21	22	−	21	20
Age (months)	11 (9 to 15)	13 (10 to 18)	.34	14 (10 to 20)	11 (9 to 14)
Male sex	12 (57)	14 (64)	.76	9 (43)	7 (35)
MUAC (mm)	119 (115 to 126)	132 (130 to 141)	<.001	113 (110 to 126)^c^	120 (117 to 120)^c^
Oedema	0 (0)	0 (0)	1.00	9 (43)^b^	0 (0)
WLZ*	−1.0 (−2.1 to −0.8)	−1.1 (−2.0 to 0.2)	.81	−2.9 (−3.5 to −2.1)^c^	−1.7 (−2.3 to −1.3)^a^
LAZ	−2.4 (−4.3 to −1.8)	−1.7 (−2.4 to −0.5)	.03	−3.3 (−3.6 to −2.3)^b^	−2.3 (−3.4 to −1.9)^a^
WAZ	−2.3 (−3.5 to −1.7)	−1.9 (−2.5 to −0.5)	.06	−3.3 (−3.8 to −2.3)^c^	−2.8 (−3.5 to −2.1)^b^
OFCAZ	−0.3 (−0.9 to 1.2)	−0.6 (−1.0 to 0.6)	.49	−0.8 (−1.7 to −0.3)	−1.2 (−1.9 to −0.5)^a^
					
Calcium** (mmol/L)	2.16 (2.04 to 2.34)	2.46 (2.37 to 2.56)	<.001	2.40 (2.19 to 2.55)	2.38 (2.30 to 2.49)
Phosphate (mmol/L)	0.95 (0.79 to 1.46)	1.92 (1.81 to 2.14)	<.001	1.57 (1.29 to 1.69)^b^	1.66 (1.49 to 2.02)
ALP (U/mL)	912 (396 to 1418)	283 (240 to 319)	<.001	188 (175 to 262)^b^	211 (176 to 297)
PTH (pg/mL)	122 (44 to 249)	26 (14 to 46)	<.001	27 (14 to 57)	27 (20 to 35)
25(OH)D (nmol/L)	19 (15 to 37)	70 (54 to 85)	<.001	53 (32 to 82)	63 (46 to 92)
1,25(OH)_2_D (pmol/L)	331 (213 to 446)	316 (277 to 384)	.57	248 (213 to 338)	316 (253 to 388)
CRP (mg/L)	2.7 (1.0 to 6.9)	2.1 (1.0 to 5.6)	.63	3.1 (1.0 to 13.5)	4.9 (2.8 to 13.1)^b^
AGP (g/L)	1.3 (0.7 to 1.6)	1.3 (1.0 to 1.5)	.84	1.9 (1.2 to 2.3)^b^	1.4 (1.2 to 1.8)
Calcium intake (mg/day)	225 (113 to 382)	117 (63 to 226)	.13	130 (78 to 245)	192 (86 to 234)
Phosphate intake (mg/day)	425 (259 to 622)	266 (154 to 505)	.14	323 (231 to 423)	394 (228 to 589)

*Note*. Values are median (interquartile range) or number (percentage) throughout. AGP = alpha‐1 acid glycoprotein; ALP = alkaline phosphatase; CRP = C‐reactive protein; LAZ = length‐for‐age; MAM = moderate acute malnutrition; MUAC = mid‐upper arm circumference; OFCAZ = occipitofrontal circumference‐for‐age; PTH = parathyroid hormone; SAM = severe acute malnutrition; WLZ = Weight‐for‐length; 25(OH)D = 25‐hydroxyvitamin D; 1,25(OH)_2_D: 1,25 dihydroxyvitamin D. For SAM and MAM, significance level of difference to control group are indicated if *p* < .05 with superscripts indicating:

a *p* < .05,

b *p* < .01,

c *p* < .001.

* Omitted in the presence of oedema.

** Calcium is adjusted for albumin as described in the methods section.

### 3.2 Relationship between anthropometric and biochemical variables among children without rickets

**Figure 3:**
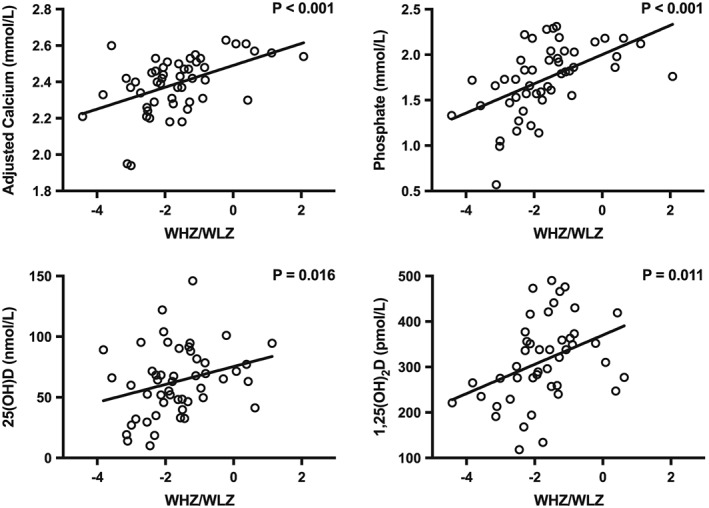
Relationship between serum adjusted calcium, phosphate, 25‐hydroxyvitamin D (25(OH)D), and plasma 1,25‐dihydroxyvitamin D (1,25(OH)_2_D) with weight‐for‐length z‐score. *p* values are for linear regression analyses corrected as described

Amongst children with SAM, the nine with kwashiorkor had a different biochemical profile from those with severe wasting (MUAC <115 mm): kwashiorkor was associated with lower albumin and ALP, and with higher 25(OH)D and CRP (supplementary Figure 2). In order to assess relationships between biochemical variables and degree of wasting (WLZ and MUAC) and stunting (LAZ), we performed linear regression excluding those children with rickets. Degree of wasting (WLZ) and absolute MUAC were associated with lower serum adjusted calcium, phosphate, 25(OH)D, and 1,25(OH)_2_D levels (Table 3, Figure 3). Stunting (adjusted for age) was also associated with lower phosphate (Table 3). Thirty‐three percent of severely wasted children (MUAC <115 mm) had a low 25(OH)D (<30 nmol/L) indicating an increased risk of vitamin D deficiency, and a further 25% was suggestive of insufficiency (30 to 50 nmol/L).

**Table 3:** Relationships between biochemical and anthropometric indices

	WLZ		MUAC		LAZ	
	Coefficient	*p*	Coefficient	*p*	Coefficient	*p*
Calcium*	0.06	<.001	0.040	.019	0.02	.296
Phosphate	0.16	<.001	0.11	.003	0.08	.048
ALP	14.9	.086	13.6	.078	7.9	.175
PTH	−7.9	.566	−7.4	.316	−7.7	.293
25(Oh)D	7.4	.016	9.3	.002	0.69	.574
1,25(Oh)_2_D	32.1	.011	2.5	.838	10.0	.299

*Note*. Outputs of linear regression analyses. Output for LAZ is adjusted for age. LAZ = length‐for‐age z‐score; MUAC = mid‐upper arm circumference; WLZ = weight‐for‐length z‐score.

* Calcium is adjusted for albumin as described in the methods section.

## 4 DISCUSSION

Vitamin D deficiency appeared to play a key role in the pathogenesis of rickets in young children in an informal settlement in Nairobi. Children with rickets tended to present with wrist widening or rachitic rosary, and acute malnutrition and developmental delay were common comorbidities. The phenotype of rickets was clinically and biochemically distinct from that caused by calcium deficiency, which is the more prevalent form reported in several other sub‐Saharan African settings (Thacher et al., 1999; Pettifor, 2004; Prentice et al., 2008; Braithwaite et al., 2016). This has important implications for treatment, for calcium supplementation alone has proven to be adequate for treatment of rickets caused by calcium deficiency. In Nairobi, it is likely that provision of supplemental vitamin D would be required (Thacher et al., 1999; Oginni et al., 2003). Furthermore, in this setting, acute malnutrition was associated with relatively low vitamin D levels, and 33% of children with SAM had absolute vitamin D levels in the deficient range.

The current study has not addressed the underlying causes of vitamin D deficiency in this setting. However, the fact that Nairobi's climate should support sufficient vitamin D biosynthesis raises important behavioural questions around sunlight exposure. Edwards et al. reported that 71% of children with rickets in another Nairobi informal settlement had less than 3 hours' sunlight exposure per week (Edwards et al., 2014). Poverty and urban living may act synergistically to limit infants' exposure to sunlight. In Mathare, the imperative for mothers to seek employment very early in the postnatal period means that infants are often looked after in indoor informal daycare facilities (“babycares”) all day. Even where infants are cared for by a parent, concerns about personal security, the use of iron sheeting without windows as the predominant building material for dwellings, and an overcrowded and overlooked built environment without open spaces all limit sunlight exposure. As well as impacting the child, this may influence maternal vitamin D status, which has important consequences for the child's vitamin D status at birth and the delivery of vitamin D in breastmilk (Salameh et al., 2016). Understanding the health and developmental consequences of growing up in sub‐Saharan African urban informal settlements is important because urbanisation is proceeding at a high rate, does not appear to be associated with reduction in poverty, and is leading to dramatic increases in the number of people living in such circumstances (Henderson et al., 2013; Awumbila, 2014).

Some children had rickets without biochemical evidence of vitamin D deficiency. Although the clinical and demographic profile of this group was similar to that of children with rickets and a low 25(OH)D, the frequency of radiologically severe disease and secondary hyperparathyroidism were lower, and biochemical evidence of increased bone turnover was less strong (Figure 2 & supplementary Table 1). Whether these children had historic, healed, or “mild” rickets is difficult to assess, but they exemplify the fact that clinical diagnosis alone fails to reflect heterogeneity in the underlying pathophysiology and intensity of disease activity in a way that may be meaningful for treatment. At present, there are no internationally accepted diagnostic criteria for rickets. Thacher and colleagues' radiographic scoring method was designed for, and validated in, children older than 12 months who were independently mobile, but in Nairobi, rickets affects very young children and is associated with a high frequency of motor developmental delay (Thacher et al., 2002; Edwards et al., 2014; Munns et al., 2016). Diagnostic criteria that apply across geography, age, and developmental status are essential prerequisites to understanding the epidemiology and measuring the burden of rickets. There is no mention of rickets in either of the World Health Organization's *Pocket Book of Hospital Care for Children*, or *IMCI (Integrated Management of Childhood Illness) Handbook*, and such data will help to improve the visibility of rickets as a significant concern in global child health (World Health Organization, 2005; World Health Organization, 2013).

The study has important limitations. We did not aim to perform population‐based screening for features of rickets, and the children recruited should be considered a convenience sample, likely biased towards more severe cases. Therefore, these data do not preclude the existence of calcium deficiency rickets in the wider population, and further studies determining the epidemiology of rickets in this setting are needed. The decision to restrict recruitment to children younger than 2 years old was pragmatic, based on the fact that this age group represented the overwhelming majority of cases referred for treatment. Although calcium deficiency rickets in other settings has mainly been found in older children, the epidemiologic comparison should not be over‐interpreted. Accurate dietetic assessment of calcium intake is very difficult in a breastfeeding population, and these data should be considered exploratory rather than definitive. When parents were completing the food frequency questionnaire, the diagnosis of rickets had been established, which increases the risk of recall bias, and although contextually‐relevant, questionnaires had not been independently validated.

There was an association between wasting and deficiencies in serum adjusted calcium, phosphate, and vitamin D. This is likely to be due to a confounding effect of poverty, which might impact directly on food security, micronutrient intake, and secondarily on sunlight exposure, as previously described. Ready‐to‐use therapeutic foods are used for the outpatient management of SAM and are manufactured to internationally accepted compositional specifications (World Health Organization, World Food Programme, United Nations System Standing Committee on Nutrition, United Nations Children's Fund, 2007). Based on their weight, most of these children would have received two to three sachets of ready‐to‐use therapeutic foods, providing high levels of calcium and phosphate, and up to 2400 IU vitamin D per day (World Health Organization et al., 2007). Although this is in line with minimum dosage recommendations for vitamin D in deficiency and rickets, the dose and duration of treatment may be suboptimal for bone health during catch‐up growth (Munns et al., 2016). SAM is associated with significant long‐term health consequences, and the development of strategies targeted at healthy growth and development in the recovery phase is a research priority (Briend & Berkley, 2016). Vitamin D deficiency is a plausible mechanism explaining at least some of the increased vulnerability to severe infectious disease in SAM, because a number of studies have indicated, it increases the risk of respiratory tract infections (Esposito & Lelii, 2015). Public health strategies to improve vitamin D levels may provide benefit beyond prevention and treatment of rickets, but should be tested in rigorous controlled trials. Solar bottle bulbs (which involve the incorporation of water‐filled plastic drink containers into the roof of a settlement) represent an innovative approach to increasing indoor sunlight exposure in urban informal settlements (http://www.literoflight.org), however the use of common polyethylene terephthalate plastic bottles means that the UVB wavelengths important for vitamin D production are filtered out (Sackey et al., 2015). Adaptation of this approach may be feasible and could provide a cheap and durable solution.

In summary, we have identified a group of young children living in an urban informal settlement in Nairobi who have rickets that appears to be predominantly caused by vitamin D deficiency and who would be likely to require vitamin D supplementation for full recovery. Development of standardised tools for diagnosis of rickets in this age group that could help establish the burden of disease should be a clinical and research priority. The apparent preponderance of vitamin D deficiency over calcium deficiency as cause of rickets in this setting prompts larger and more detailed epidemiologic studies in this and other settings.

## CONFLICTS OF INTEREST

The authors declare that they have no conflicts of interest.

## CONTRIBUTIONS

KDJJ, CUH, BHK, AP, JAB designed the study. KDJJ, CUH, CM, HSN were involved in clinical aspects of the study. M. K. coordinated dietary assessment. KDJJ, LC, IS, AP were involved in laboratory aspects of the study. KDJJ, JAB analysed the data. KDJJ wrote the first draft of the manuscript, all authors contributed to subsequent drafts and approved the final copy.

## Supporting information

Supplementary Table 1: Clinical features in children with rickets with or without low 25(OH)D
Supplementary Table 2: Relationships between biochemical and anthropometric indices adjusted for inflammatory activation
Supplementary Figure 1: Relative contribution of low vitamin D to rickets was not a function of age at diagnosis.
Supplementary Figure 2: Biochemical indices in severe wasting and kwashiorkor
