# Supplementary material for: Vitamin D deficiency causes rickets in an urban informal settlement in Kenya and is associated with malnutrition
Source: Matern Child Nutr. 2017 May 3;14(1):e12452. doi: 10.1111/mcn.12452 (PMC5763407; doi:10.1111/mcn.12452)
Supplement: Supplementary file 1 — Supplementary Table 1: Clinical features in children with rickets with or without low 25(OH)D Supplementary Table 2: Relationships between biochemical and anthropometric indices adjusted for inflammatory activation Supplementary Figure 1: Relative contribution of low vitamin D to rickets was not a function of age at diagnosis. Supplementary Figure 2: Biochemical indices in severe wasting and kwashiorkor [file MCN-14-e12452-s001.docx]

**SUPPLEMENTARY MATERIAL FOR REVIEW & ONLINE PUBLICATION**

**Supplementary Table 1: Clinical features in children with rickets with or without low 25(OH)D**

|  | | Low 25(OH)D | Normal 25(OH)D | P |
| --- | --- | --- | --- | --- |
| n | | 14 | 6 | - |
| Age (months) | | 12 (10 to 14) | 10 (8 to 17) | 0.62 |
| Male sex | | 6 (43) | 5 (83) | 0.16 |
| Proportion with Thacher Grade 2 wrist radiographic features | | 10* (83) | 2 (33) | 0.11 |
| Major features | Wrist widening | 14 (100) | 6 (100) | 1.00 |
|  | Rachitic rosary | 14 (100) | 4 (67) | 0.08 |
|  | Swollen knees | 3 (21) | 1 (17) | 1.00 |
|  | Bow legs | 0 (0) | 2 (33) | 0.08 |
|  | Bone pain on walking | 1 (7) | 0 (0) | 1.00 |
| Minor features | Open fontanelle | 12 (86) | 6 (100) | 1.00 |
|  | Double malleoli | 7 (50) | 3 (50) | 1.00 |
|  | Harrison’s groove | 6 (43) | 4 (67) | 0.63 |
|  | Lower arm bending | 3 (21) | 0 (0) | 0.52 |
|  | Developmental delay | 8 (57) | 2 (33) | 0.63 |
| Nutritional status | SAM | 4 (29) | 0 (0) | 0.27 |
|  | MAM | 5 (36) | 5 (83) | 0.14 |
|  | Stunting | 10 (71) | 3 (50) | 0.61 |
| ALP (U/L) | | 1356 (896 to 1589) | 355 (315 to 708) | 0.005 |
| PTH (pg/mL) | | 181 (112 to 295) | 25 (13 to 44) | 0.002 |
| 1,25(OH)_2_D (pmol/L) | | 306 (184 to 430) | 438 (351 to 447) | 0.20 |
| Calcium intake (mg/day) | | 207 (126 to 372) | 225 (134 to 363) | 0.92 |

**Supplementary Table 1:** Children with rickets who had 25(OH)D <30 nmol/L or ≥30 nmol/L. Values are median (interquartile range) or number (percentage) throughout. Thacher Grade 2 indicates the presence of the more severe radiological features of concave metaphyseal cupping and frayed margins ([Thacher et al., 2000](#_ENREF_27)). * of 12 participants with low 25(OH)D for whom radiographs were available for review. Note one child with rickets was excluded because no 25(OH)D result was available.

**Supplementary Table 2: Relationships between biochemical and anthropometric indices adjusted for inflammatory activation**

|  | WLZ | | MUAC | | LAZ | |
| --- | --- | --- | --- | --- | --- | --- |
|  | Coefficient | P | Coefficient | P | Coefficient | P |
| Calcium | 0.06 | 0.001 | 0.04 | 0.023 | 0.02 | 0.364 |
| Phosphate | 0.19 | <0.001 | 0.12 | 0.002 | 0.08 | 0.012 |
| ALP | -2.8 | 0.488 | -6.7 | 0.681 | 2.5 | 0.212 |
| PTH | -9.4 | 0.371 | -8.5 | 0.190 | -6.7 | 0.440 |
| 25(OH)D | 7.4 | 0.016 | 9.7 | 0.002 | 0.52 | 0.653 |
| 1,25(OH)_2_D | 33.3 | 0.010 | 1.7 | 0.896 | 10.2 | 0.299 |

**Supplementary Table 2:** Outputs of linear regression analyses, all adjusted for C-reactive protein and alpha-1 acid glycoprotein. Output for LAZ is also adjusted for age. MUAC: mid-upper arm circumference; WLZ: weight-for-length z-score; LAZ: length-for-age z-score

**Supplementary Figure 1: Relative contribution of low vitamin D to rickets was not a function of age at diagnosis.**

**Supplementary Figure 2: Biochemical indices in severe wasting and kwashiorkor**

**Supplementary Figure 2:** Differences between children diagnosed with severe acute malnutrition on the basis of severe wasting (mid-upper arm circumference <115 mm without oedema) or Kwashiorkor (bilateral lower limb oedema). *P<0.05; **P<0.01; ***P<0.001. Calcium is corrected for albumin as described in the text. Horizontal lines indicate median. ALP: alkaline phosphatase; PTH: parathyroid hormone; 25(OH)D: 25-hydroxyvitamin D; 1,25(OH)_2_D: 1,25-dihydroxyvitamin D; CRP: C-reactive protein; AGP: alpha-1 acid glycoprotein.
